# Neuronal Glial Crosstalk: Specific and Shared Mechanisms in Alzheimer’s Disease

**DOI:** 10.3390/brainsci12010075

**Published:** 2022-01-03

**Authors:** Vishal Chavda, Kavita Singh, Vimal Patel, Meerambika Mishra, Awdhesh Kumar Mishra

**Affiliations:** 1 Division of Anesthesia, Dreamzz IVF Center and Women’s Care Hospital, Ahmedabad 382350, Gujarat, India; chavdavishal2@gmail.com; 2 Centre for Translational Research, Jiwaji University, Gwalior 474011, Madhya Pradesh, India; kavita.kushwaha786@gmail.com; 3 Department of Pharmaceutics, Nirma University, Ahmedabad 382481, Gujarat, India; email2vimal.patel@gmail.com; 4 Department of Infectious Diseases and Pathology, University of Florida, Gainesville, FL 32611, USA; 5 Department of Biotechnology, Yeungnam University, Gyeongsan 38541, Gyeongbuk, Korea

**Keywords:** neuroglia, microglia, astrocytes, oligodendrocytes

## Abstract

The human brain maintains billions of neurons functional across the lifespan of the individual. The glial, supportive cells of the brain are indispensable to neuron elasticity. They undergo various states (active, reactive, macrophage, primed, resting) and carefully impose either quick repair or the cleaning of injured neurons to avoid damage extension. Identifying the failure of these interactions involving the relation of the input of glial cells to the inception and/or progression of chronic neurodegenerative diseases (ND) is crucial in identifying therapeutic options, given the well-built neuro-immune module of these diseases. In the present review, we scrutinize different interactions and important factors including direct cell–cell contact, intervention by the CD200 system, various receptors present on their surfaces, CXC3RI and TREM2, and chemokines and cytokines with special reference to Alzheimer’s disease (AD). The present review of the available literature will elucidate the contribution of microglia and astrocytes to the pathophysiology of AD, thus evidencing glial cells as obligatory transducers of pathology and superlative targets for interference.

## 1. Introduction

Neuron–glia crosstalk has been an appealing issue for neuroscientists around the globe due to escalating conditions of neurodegenerative diseases in the elderly population [1]. Neurodegenerative disease comprises both systems of the body, i.e., the central and peripheral nervous system. The most prevalent neurodegenerative disease of the central nervous system is known as Alzheimer’s disease (AD) and has been a burning issue of investigation for decades now. Due to its large societal impact, neuroscientists have explored highly advanced techniques to understand its pathogenesis, pathobiochemistry, and neuron–glia interactions, which might be a new therapeutic platform to control these deadly diseases [2,3]. Alzheimer’s poses a major health challenge when we think of mental illness. According to the World Health Organization, reports suggest that this type of dementia will increase threefold by 2050 [4,5]. Accumulating evidence suggests that alterations of neuron-glia interactions are associated with the development of neurodegenerative diseases referred to as “tauopathies” [6]. Astrocytes perform significant functions including in synapse formation and plasticity, energetic and redox metabolism, and the synaptic homeostasis of neurotransmitters and ions [7]. Microglia represent the immune system of the brain and therefore are critically involved in various injuries and inflammatory diseases. Oligodendrocytes have a role in the regulation of steroid synthesis, which is important for neuroprotection against degeneration [8]. A glia-mediated inflammatory response is implicated in remarkable changes in the activity of neuritic plaque-associated astrocytes and microglia, and the connection between glial activation and neuronal damage or repair has been suggested [9]. In addition, the functional relationship between neurons, glial cells, and vascular cells within so-called neurovascular units is dramatically compromised in AD.

Therefore, the importance of alterations in synergistic interactions between cells (neurons, microglia, and astrocytes) in the pathogenesis of this neurodegenerative disorder has been suggested [10]. Furthermore, an understanding of the molecular mechanisms of neuron–glia interactions in AD would give us novel diagnostic and therapeutic strategies. Neurodegenerative diseases are mainly characterized by neuronal death and loss of neuronal activity in an age-dependent manner. Other common characteristics for neurodegenerative diseases include abnormal thinking, emotional imbalance, behavioral changes, and social disturbances [11]. The pathogenesis of almost all neurodegenerative diseases is characterized by the buildup of an atypical and anomalous form of protein that is noxious to neuronal survival [12,13,14,15]. The important risk factors for neurodegeneration in the CNS include genetic as well as environmental stressors [16]. Despite the large improvement in neuro-techniques and the advancement in histological examinations in neurodegenerative diseases, there is still no major cure for the treatment of neurodegeneration as they are only symptomatic for a limited duration within a lifespan [17].

Alzheimer’s is the most fatal non-curable neurodegenerative disease, and the most lethal form of dementia, affecting more than 50 million people globally as reported in 2017; this is estimated to double every 20 years. The major characteristic features of AD include age-dependent dementia, learning and memory loss, imbalance in thoughts, low thinking and decision-making ability, mood disorders, delusions, and cognitive decline evidenced in overall personality alterations [18,19]. Several explicative theories have been proposed, but the precise pathophysiological mechanisms are vague. The primary neuropathology of AD includes neurotic amyloid plaques and neurofibrillary tangles (NFTs) [20]. The foremost symptom of AD is a loss of neural circuit integrity. In the manifestation of this danger sign, neurons do work with glial cells coordinately. In the central nervous system (CNS), the reciprocal relationship between neurons and astrocytes is crucial for signaling, extracellular ion homeostasis, energy metabolism, and neuroprotection [21]. The intricate unbalanced and perturbed interactions of astrocytes and neurons are emerging strongly in the study of AD [22].

As stated above, astrocytes and microglia exert their primary functions on neurons, and much research addresses the dyadic interactions: microglia–neuron and astrocyte–neuron [23]. It is also timely to consider how microglia and astrocytes signal to each other, to obtain a more comprehensive account of how their behavior is regulated in the complex context of CNS injury or disease [24]. This review takes the approach of briefly introducing each cell type in relation to its interactions with neurons, followed by a series of diagrams illustrating how microglia and astrocytes can communicate. Finally, these interactions will be placed in the setting of varied CNS disorders. In each circumstance, the relevant outcome of astrocyte–microglial communication will be the health of the individual neuron or the integrity of the neural circuit.

The connection between astrocytes and microglia interaction is still unexplored, although neuroinflammation is a key process in understanding the various CNS pathologies, and it is well accepted that both these cell types are involved in very close, dynamic, and continuous crosstalk. This bidirectional communication is crucial in resting, activated, and aged phenotypes, but it the mechanism underlying this is only starting to be explored [25,26].

## 2. Microglia and Neuron Interaction in Alzheimer

Microglia are prime immune cells of myeloid origin in the brain. Microglial cells are the very first cells which energetically encounter stressors and respond to proinflammatory mediators through a process known as microglia activation [27]. Afterwards, astrocytes move to injured or inflamed sites to display a beneficial profile by the secretion of IL-10 followed by the upregulated TGF-β secretion by astrocytes. TGF-β plays a neuroprotective role and restricts inflammation, strengthening the non-inflammatory microglia M2 phenotype [28]. In AD, TGF-β was reported to protect against the toxic effects of amyloid accumulation in murine cultures [29]. However, if the neuroinflammation is not executed, the microglia cells transform into reactive forms through a process known as microgliosis and introduce the complement compnent1q protein (C1q), TNF-α, and IL-1β into the brain.

**Figure 1:**
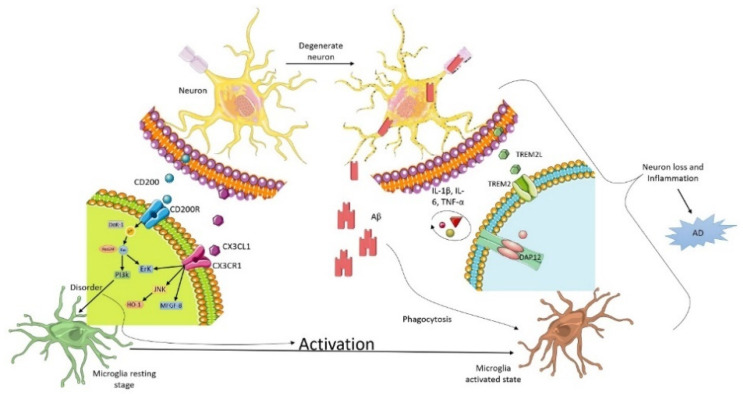
Microglia and neuron communication in AD pathology.

Microglia as components of the innate immune system act in response to various environmental stimulants including the amyloid-β(Aβ) protein, which accumulates and is toxic to the brain as it downregulates the Aβ metabolism, causing phagocytosis by microglia, and is responsibleto large extent for AD pathogenesis [30]. Upon the encounter with the stressor (injury, infection, malnutrition), microglia undergo morphological alterations, leading to upregulated cell proliferation, migration, macrophagic activity, the stimulation of the NLRP3 inflammasome, and eventually a blizzard of pro-inflammatory mediators [31,32], although the correlation of proinflammatory cytokines with aging following CNS injury is still a subject of research. Figure 1 represents microglia and neuron communication in AD pathology.

## 3. Triggering Receptors Expressed on Myeloid Cell-2 (TREM2) Receptor

Microglial cells are housekeeping cells and support neuronal function and survival in the central and peripheral nervous system [33]. They are the most dynamic and notorious part of the neuro-immune cells and thus they are actively involved in the immune regulation of the various protein parts. Therefore, we sought to compile the role of microglia cells in the central part and thus this review provides the mechanism of action occurring between neurons and microglial cells in the development and progression of neurodegenerative diseases.

For the continuous maintenance of dynamic and consistent neuronal signaling, see [34]. Microglia acquire their name from “glia”, meaning glue, which describes their main function, i.e., to act as nerve glue or nerve cement. Some of the latest research evidences the crucial role of glial cells in many brain diseases including AD, Parkinson’s disease, multiple sclerosis, and amyotrophic lateral sclerosis [35]. Categorically speaking, glial cells are of three types: microglia, astrocytes, and oligodendrocytes (OLS). Moreover, there is another newly discovered form, neural progenitor cells, expressing chondrotitin sulphate proteoglycan 4, which are called oligodendrocyte progenitor cells or NG2-glia [36].

It has been reported that amyloid plaques in AD patients are related to microglia because the pro- and anti-inflammatory types are based on the decline in numerous homeostatic genes and increase of AD-associated risk factors [37,38,39]. Microglial function is primarily regulated and controlled by triggering a receptor protein known as TREM2 that is expressed on myeloid cells. There are two main types of TREM2 signaling pathway, the first of which controls the macrophagic activity of microglial cells to eradicate the waste cellular debris and Aβ plaques and thus provide neuroprotection, whereas the other signaling mechanism results in overturning the TREM2 which further results in starting the down-signaling mechanism. This eventually leads to the downregulation of cytokine production followed by the stimulation of inflammatory pathways and neuronal loss [40]. 

Microglia have phenotypic switching capabilities, which means they can change their shape and functional features in response to injury, neurodegeneration, or stress. Furthermore, they go from a resting state to an active one where they retract their thin long process into voluminous and thick filopodia. This active form is incompetent in regulating homeostasis in the CNS and moreover they delineate their phagocyte function [41]. During the initial onset of αβ protein in AD, microglia cells eliminate these depositions through macrophagic activity, but in later stages when the disease enhances the proinflammatory mediators (cytokines and chemokines) which are secreted by microglial cells, they decrease the expression of neuroprotective genes against AD.

Microglia tend to remain immunologically silent until triggered by an immune challenge in ahealthy brain. This homeostatic microglial function is supported by neuronal immunomodulators like CX3CL1 and CD200, which act as the ligand for their receptors located on microglia. This homeostatic function of microglial is disturbed in neurodegenerative conditions [42].

### 3.1. CD200

CD200 is a type I membrane glycoprotein that is located on the neuronal surface in the rodent brain, and binds to the myeloid cell receptors present on the microglia surface CD200 receptor (CD200R) [43]. A decline in CD200 and CD200R has been reported in mouse models of chronic and acute neuroinflammation, suggesting a deregulation of this mechanism in neurodegenerative diseases [44]. The binding of neural CD200 and its receptor CD200R have been reported to be reduced in human AD samples and in neuroinflammation mouse models, suggesting a distortion in this mechanism. Their ligation attenuates microglia physiology in the AD tissues [45,46]. A CD200-knockout mouse model presents symptoms of glial activation and an upregulated inflammatory profile following brain injury and experimentally induced autoimmune encephalomyelitis, recommending the crucial role of CD200 in the immune function performed by microglia in CNS [47].

### 3.2. CX3XL1

Fractalkine or neurotactin is secreted by neurons in the CNS, whereas its receptor CX3XR1 is located on the microglial surface [48]. Genetic modifications of CX3CR1 have demonstrated an enhanced microglial population in various models of neuroinflammation, suggesting that the CX3CL1/CX3CR1 interaction maintains the microglial population in homeostasis in a normal physiological state [49]. In various models mimicking AD pathology, the genetic mutation of CX3CAR1 has been found to produce adverse effects corresponding to the conditions of the AD model. CXC3CR1 is associated with the transcriptomic signature status of microglial cells depicting normal conditions, but in AD pathology, the downregulation of CXC3R1 has been reported recently, revealing disturbances in the homeostasis of the CNS [38,50,51,52,53,54]. Moreover, this information is evidence that a dysregulated neuro-glia interaction is now a hallmark of neurodegenerative disorders and might cause the activated and probably deleterious and distorted phenotype of microglia in AD.

### 3.3. Extra-Internal Neuronal Signaling Pathways

The functional units of neuronal components such as neurons, astrocytes, and microglia comprise pre-and postsynaptic clefts termed “tripartite synapses”, where the whole of neurotransmission and neuromodulation takes places [55]. The densities of tripartite synapses vary in different brain areas, which suggests a diverse pattern of neuronal signaling. Estimation of tripartite synapses would be harder during sensory stimulation and neuronal activity [56]. Neuromodulation and neurotransmission occur in response to the astrocytes releasing Ca^2+^ induced by triphosphates through the activation of G-protein coupled receptors (GPCR) [57]. Nedergaard et al. suggested that the administration of cocktail neuromodulators increases the K^+^ level, enhancing synaptic activity in neuronal and non-neuronal networks when administered to cortical brain slices [58]. Neuromodulators such as Ach, serotonin, noradrenaline, histamine, and dopamine promote synchronous excitatory and oscillatory signals between astrocytes and neurons, byaltering the K^+^ clearance rate [59,60]. The corticotrophin-releasing hormone (CRH) neurons in the hypothalamus are activated by norepinephrine, which further activates astrocytes through dendritic signaling and releases ATP in presynaptic neurons [61]. Epinephrine promotes ATPase activity, inducing astrocytic metabolic disturbance leading to behavioral changes [62]. The influx of K^+^ in glial cells is made to fluctuate by the serotonin [63]. These break down adrenergic and serotonin pathways inside the locus coeruleus and raphe nuclei in rodents. The perceived neurodegeneration and dysregulation of the glial–neuron communication plays a vital role in AD pathologies and proression [64,65]. 

### 3.4. Astrocytes and Neuron Interaction in AD

Astrocytes are star-shaped glial cells covering the entire CNS thatsupply essential physiological support for neurons. Indeed, neurons fail to function without the critical functions provided by astrocytes in the healthy CNS. The maintenance of the blood–brain barrier (BBB), modulation of synapses, and supply of trophic and metabolic support to neurons are basic normal physiological functions of astrocytes [66,67,68]. The neurons perform excitatory and inhibitory synaptic activity, facilitate electrical and chemical transmission, enable synaptic plasticity, and allow circuitry function and cognition. In AD, neurons and astrocytes show noxious interactions. Catastrophic cascading effects on neurons are imparted by astrocytes while responding to a toxic stimulus by modifying their gene expression, morphology, proteomes, and secretome. Astrogliosis, beneficial and impaired clearance of amyloid and tau proteins, the secretion of neurotoxic and proinflammatory agents, synaptic phagocytosis, impaired synaptic homeostasis, and BBB disruption are remarkable astrocytic toxic changes manifested in early AD. Synaptic dysfunction causing amyloid and/or tau deposition isone of the most unfavorable events occurring in early AD [22].

Astrocytes are primarily essential for processes including the maintenance of a homeostatic state, the uptake and recycling of neurotransmitters, the release of gliotransmitters, facilitating neuro-energetics, participating in cerebral inflammation, the modulation of synaptic activity, and the maintenance of the BBB and ionic balance [69,70,71]. In contrast to neurons, astroglia are homogenic physiologically important cells, expressing distinct molecular markers such as GFAP, calcium-binding protein S100B, glutamine synthetase, and Aldh1L1 [72]. Owing to the wide array of physiological characteristics, astrocytic dysfunction remains an indispensable agonist of the cytotoxic cascade and an antagonist of glial–neuronal and glial–vascular signaling in neurological disorders [73]. In line with this, it should be noted that several changes in astrocyte function have been observed in the brains of AD patients, animal models, and in vitro studies. An Aβ deposit, the primary cause of AD, interferes with astrocytic functions involving glial transmission, neurotransmitter uptake, and calcium signaling. The changes in astrocytes induce other downstream pathological events in AD, indicating astroglia dysfunction as an early cause of the following sequelae in AD. The critical coordinated contribution of astrocytes and neurons has led to them being an attractive target for the study of AD pathogenesis and therapeutics [19,74].

The leading pathological hallmarks of AD are the appearance of extracellular Aβ plaques, the formation of intraneuronal neurofibrillary tangles primarily composed of hyperphosphorylated tau, and brain atrophy, along with enhanced cerebral neuroinflammation [75,76]. The Aβ protein stimulates several different cell receptors and regulatory signaling cascades in astrocytes. The neural inflammation is majorly ameliorated by the activation of the NF-κB pathway [77] and the advanced glycation end product receptor. Both pathway and receptor activation trigger the transcription of pro-inflammatory mediators in astrocytes [78]. The proinflammatory agents successively encourage cytotoxicity, cellular damage, or even stimulate the production of Aβ in astrocytes. Additionally, Aβ induces mitochondrial dysfunction and excitotoxicity by means of escalated oxidative stress, increased production of reactive oxygen and nitrogen species (ROS and RNS), high influx of C^2+^ ions, elevated levels of NADPH oxidase (NOX), amplified NF-κB signaling, and excessive glutamate uptake in astrocytes. The glutamine–glutamate/GABA cycle is a leading metabolic pathway implicating neurons and astrocytes in a coordinated fashion [79]. The cycle includes the release of glutamine or GABA from astrocytes to glutamatergic and GABAergic neurons. In AD, the astrocytic transfer of GABA as well as the GABA–glutamine cycle appears to be dysregulated [60,80]. Furthermore, astrocytes are implicated in producing, degrading, and removing Aβ via the expression of apolipoprotein E (APOE). The astrocytic molecule APOE plays a vital role in the initial deposition of Aβ, remodeling of Aβ into dense core plaques, and amyloid clearance. Astrocytic APOE expression is also able to influence neuronal death in AD. While comparing APOE2, APOE3, and APOE4 isoforms in AD mouse models, APOE4 exacerbates plaque aggregation [81]. The APOE null mice demonstrated decreased plaque deposition [82], emphasizing the dark role of astrocytes in AD pathogenesis—more production and inoculation of amyloid proteins in neurons than amyloid clearance [22]. In general, astrocytes get accumulated around the neurons but fail to perform a neuroprotective Aβ/tau debris-clearing function. In fact, they release chemicals that cause chronic neuroinflammation and further damage the neurons they are meant to protect. Furthermore, the AD brain becomes rich in astrocytic S100β, a protein that significantly participates in periplaque etiology, enhancing dystrophic neurites within plaques [83,84]. Hence, astrocyte–neuron interaction exhibits pathological events such as neuroinflammation, excitotoxicity, mitochondrial dysfunction [74].

The neuron–astrocytic glutamine-GABA cycle is important for the replenishment of the neuronal glutamate pool for neurotransmission and aids in the regulation of energy metabolism, excitability, and signal transduction within and between the astrocytic and neuronal networks [85]. The synaptically released glutamate, intracellular Ca^2+^ waves, and paracrine interactions mediated by glutamate, ATP and cyclic ADP-ribose are some of the excitatory signals in the brain, which are transmitted at a distance through simultaneously interactive networks of neurons and astrocytes [21,86]. The astrocytic excitatory amino acid transporters uptake neuronal released synaptic glutamate to inhibit the accumulation of glutamate in the synaptic space, and thereby preventing excess activation of neuronal glutamate receptors and excitotoxicity, to achieve neuroprotection [87,88]. Recently, in-vivo and in-vitro studies highlighted that to confer neuroprotection, reactive astrocytes highly express an insulin-degrading enzyme, astrocytic matrix metalloproteinase, to degrade Aβ plaques [89,90,91]. The resistant mechanism against cerebral oxidative stress is the astrocytic production of glutathione—an antioxidant to protect neurons [92]. Besides endothelial cells, astrocytic foot ends can also supply glucose to neurons via GLUT 3 for metabolic support [93]. Furthermore, during synaptic activity, the astrocyte–neuron lactate shuttle mechanism has been proposed to be crucial for metabolic support of neurons [94]. Therefore, the astrocyte–neuron interplay serves as a vital component for the maintenance of normal physiological functions in the CNS and can also assist pathological events.

### 3.5. Contradiction of Metabolic Lactate Transition between Astrocytes and Neurons

Lactate serves as an alternative source of energy substrate in various circumstances in the brain in place of glucose. Since lactate cannot directly diffuse across the BBB, it (lactate) is produced inside the brain and transported between the cells and utilized in various neuronal activities. Researchers provided two opposed paradigm of lactate metabolism. The astrocyte–neuron lactate shuttle (ANLS) hypothesis was first published in 1994 by Pellerin and their colleagues. They claimed that lactate is produced by astrocytes and sunk to the neurons [95]. As astrocytes increase their glucose intake in response to increased neuronal activity, the rate of glycolysis and lactate released into the extracellular space increases. Increased neuronal activity results in more glutamate being released into the synapse from presynaptic vesicles. Astroglial glutamate transporters detect and absorb excess glutamate. In a 1:1 stoichiometric ratio, glutamate uptake causes glucose uptake by astrocytes. The α2 subunit of the Na^+^/K^+^ -ATPase is activated by a greater Na^+^ concentration in astrocytes, which stimulates glycolysis [96]. Bak and colleagues, in contrast to the ANLS hypothesis, propose that oxidative lactate metabolism in neurons happens only during repolarization (and the time between depolarizations), not during neurotransmission activity. According to their model, increased neurotransmission may not increase lactate oxidative metabolism; instead, it may decrease due to a depolarization-induced increase in intracellular Ca^2+^ concentration and a putative limitation of the malate–aspartate shuttle (MAS), which transfers reducing equivalents from NADH produced during glycolysis into mitochondria [97]. Most neurodegenerative diseases, as well as any other unfavorable alterations in the brain, are thought to cause significant changes in the ANLS, resulting in neurometabolic coupling abnormalities.

### 3.6. Calcium Homeostasis

**Figure 2:**
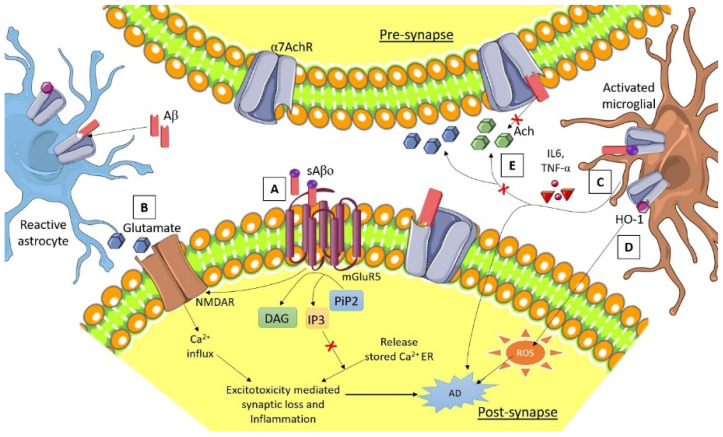
Astrocyte and neuron interaction and regulation of calcium homeostasis.

Intercellular and intracellular Ca^2+^ transport is the basic conversation of astrocytes, by which astrocytes communicate with the neuron and among themselves, causing the astrocyte to carry out a controlled messenger distribution to initiate differential response [98]. Intracellular Ca^2+^ release by the endoplasmic reticulum after the activation of inositol 1,4,5-triphosphate receptor expression releases stimulating gliotransmitters such as glutamate, GABA, D-serine, and ATP [99]. The release of glutamate activates tripartite synaptic neurotransmission within brain cells [100]. Significant reports have suggested AD pathologies associated with Ca^2+^ dysregulation [101]. The functioning of Ca^2+^ dependent synapse and Ca^2+^ homeostasis was regulated by both presenilins and amyloid precursor protein (APP) [102]. Disturbance of the Ca^2+^ signaling between the neurons and glial cell Ca^2+^ homeostasis was stimulated by the Aβ-aggregation-induced neurotoxicity [103]. Glutamate associated excitotoxicity and the activation of extra synaptic receptors dysregulate astrocytic Ca^2+^, leading to the generation of neuronal oxidative stress by the production of ROS [104]; see Figure 2, denoting astrocyte and neuron interaction and the regulation of calcium homeostasis.

There are many astrocytic membrane receptors such as metabotropic glutamate receptor type-5 (mGluR5) and calcium-permeable α7 nicotinic acetylcholine receptor (α7nAChRs) that are mediating responses regarding the altering of Aβ-induced physiology [105,106].

### 3.7. α7 Nicotinic Acetylcholine Receptor (α7nAChRs) Synaptic Function in AD Pathology

Alarge expression of α7nAChRs subunits on the astrocytic cells has been found in the cortex and hippocampal region in both AD patients and APP-derived AD animal models; this suggests that activated astrocytes are involved in the Aβ metabolism [107]. The elevated levels of Aβ discretely control presynaptic and synaptic activities, and concentration-dependently inhibit or active the α7nAChRs [108]. The evidence suggests that α7nAChR agonist agents significantly inhibit aggregation and enhanced phagocytosis of Aβ [109]. α7nAChRs activation regulate the production of proinflammatory cytokines in astrocytes such as IL-6 and TNF-α, which induce neuroinflammation [110]. Sadigh-E et al. have suggested that there is a close relation between neuroinflammation, neurodegeneration, and Aβ deposition in AD early stage [111]. α7nAChRs agonist treatment reduces the production of proinflammatory cytokines in astrocytes and decreases neuroinflammation in AD patient [108]. Interaction between Aβ and astrocytic α7nAChRs release glutamate, which causes the loss of synaptic neurons, excitotoxicity, reduced synaptic plasticity, and cognition [112]. Even microglial activation by the α7nAChRs releases intracellular Ca^2+^ and stimulates the phospholipase C signaling pathway, which reduces the neuroinflammation in AD [78]. Heme oxygenase-1 (HO-1) expression is also induced by α7nAChRs in microglia, which induces ROS-mediated neuroinflammation in AD [113].

### 3.8. Microglia and Astrocytes

The most imperative and insidious proinflammatory cytokine produced by glial cells (microglia and astrocytes) during inflammatory conditions is TNF-α [114]. Negatively, it affects mainly oligodendrocytes and causes demyelination (loss of myelin protein). For instances, Lipopolysaccharide (LPS) exposure to mixed cultures of glial cells secretes a significant amount of TNF-α, which causes systemic inflammation [115,116]. TNF-α activates the immune response through affecting cytokines secretion against systemic infection (stress) as well as enhancing the amino-3-hydroxy-5-methyl-4-isoxazolepropionic acid receptor (AMPA receptor) production and decreasing the GABA receptors. Therefore, TNF-α plays a significant role in stimulating synapses activators and leads to maximum neural activity [25,117].

### 3.9. Oligodendrocytes

Oligodendrocytes have been known as the myelinating glial cells of the CNS [118]. Overall, the homeostasis of the myelin content in the CNS is dependent upon the crosstalk between oligodendrocytes, astrocytes, and microglia. Myelin debris and the unwanted myelin population need to be engulfed by the phagocytosis action via microglia [119]. Although the neuroinflammation caused by any stress could damage the ability of remyelination and increase the decline in myelin-associated proteins [120,121,122,123,124]. Demyelinating lesions in neonatal-LPS infected Sprague Dawley rats from 3 to 24 month substantiate the neuronal and white matter injury following neonatal LPS infusion and mimic septicemia and cystic fibrosis alterations [125,126]. The preferential loss of myelin oligodendrocyte glycoprotein in the cortical region in all the age groups studied is equivalent to the demyelinating lesions of multiple sclerosis, as reported previously [127,128].

Oligodendrocytes progenitors (OPCs) give rise to oligodendrocytes which have an immense power of proliferation and migration differentiation for the recruitment of myelinating oligodendrocytes. OPCs are demonstrated by the high expression of A2B5, the receptors of PDGF alpha (PDGFRαR) and the NG2 proteoglycan. Various transcription factors including Olig1, Olig2, Mash, Myt1, Nkx 2.2, and Sox 10, and molecules like IGF-1, FGF2, CNTF, and thyroid hormone T3 regulate the specification and differentiation of OPCs [129]. The loss of myelin, called demyelination, which often appears as demyelinating lesions is usually caused by inflammation-causing stressors which also compromise the efficacy of the myelination [130]. Therefore, remyelination is followed by restoration of myeline sheaths to axons, thus shielding them from disintegration.

### 3.10. Mast Cell and Microglial Interactions in AD

**Figure 3:**
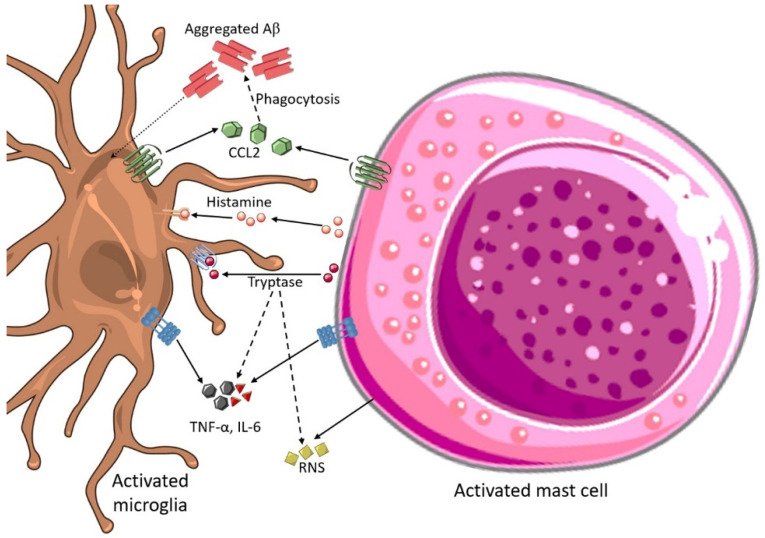
Activated microglia involved in the communication with mast cells in AD pathology.

Mast cells are intimate to the microglia found in the brain, smoothing dynamic interactions. The complex crosstalk within the brain cells and mast cells happens in multidirectional interactions by using surface adhesion molecules or receptors termed mast cell mediator [131]. These mediators’ mast cells initialize neuroinflammation and express inhibitory and costimulatory surface mediators which further communicate with immune cells such as B-cells and T-cells, functioning as a primary association between adaptive and innate immunity [132]. The activation of microglial cells and phenotypic alterations mainly occur after histamine secretion by activated mast cells in the brain [133]. Figure 3 shows how activated microglia are involved in the communication with mast cells in AD pathology.

Murine N9 microglial cortical primary cultured microglia release RNS, IL-6, and TNF-α after activated by exogenous introduction of histamine [134]. Histamine has the dual role of stimulating and inhibiting microglial migration by controlling inflammatory responses [135]. Chemokine (C-C motif) ligand-2 (CCL2), also called MCP-1, are mast cell-derived neuro-inflammatory cytokines released from mast cells influence microglial activation [136]. The concept that mast cells could regulate microglial phagocytosis is supported by the expression of CCL2-altered Aβ phagocytosis [137]. Elevated CCL2 levels are associated with AD progression and cognitive decline [138]. A large number of tryptase-positive mast cells are gathered close to Aβ plaques as shown in the histopathology of an AD autopsy brain [139]. Similar kinds of mast-cell accumulation patterns were observed in the cortex and hippocampus before Aβ deposition in transgenic mice-borne AD. The activation of Panx1 and Cx43 hemichannels was observed in brain mast cells treated with Aβ peptide in vitro, the consequence of histamine release and enhanced Ca^2+^ influx [140,141].

## 4. Conclusions

Various clinical drug trials of AD drugs have failed in the near past leading neuroscientists to reflect on other ventures which involve neuroinflammation and the connection of neurons with other cells, mainly microglia and astrocytes. Therefore, it is indisputable that microglial and astrocytes should be taken as one of the main therapeutic interventions for the development of new medicines and analytical and clinical therapy for Alzheimer’s. The detailed inspection of the interactions of astrogliosis and microgliosis will offer mechanistic insights into the progression of Alzheimer’s and useful novel therapeutic and clinical markers at the early stages of AD. Collectively, the current research involving astrogliosis and microgliosis in AD pathogenesis has provided a plethora of recently developed models to pursue this area.

## Data Availability

Not applicable.
